# Engineering of Ribosome-inactivating Proteins for Improving Pharmacological Properties

**DOI:** 10.3390/toxins12030167

**Published:** 2020-03-09

**Authors:** Jia-Qi Lu, Zhen-Ning Zhu, Yong-Tang Zheng, Pang-Chui Shaw

**Affiliations:** 1 School of Life Sciences, The Chinese University of Hong Kong, Shatin, N.T., Hong Kong 99077, China; lujq@link.cuhk.edu.hk (J.-Q.L.); janet.chuk@gmail.com (Z.-N.Z.); 2 Key Laboratory of Animal Models and Human Disease Mechanisms, National Kunming High level Biosafety Research Center for Non-human Primates, Kunming Institute of Zoology, Chinese Academy of Sciences, Kunming 650223, Yunnan, China; zhengyt@mail.kiz.ac.cn

**Keywords:** ribosome inactivating protein, therapeutic applications, immunotoxin, anti-HIV, anti-cancer

## Abstract

Ribosome-inactivating proteins (RIPs) are N-glycosidases, which depurinate a specific adenine residue in the conserved α-sarcin/ricin loop (α-SRL) of rRNA. This loop is important for anchoring elongation factor (EF-G for prokaryote or eEF2 for eukaryote) in mRNA translocation. Translation is inhibited after the attack. RIPs therefore may have been applied for anti-cancer, and anti-virus and other therapeutic applications. The main obstacles of treatment with RIPs include short plasma half-life, non-selective cytotoxicity and antigenicity. This review focuses on the strategies used to improve the pharmacological properties of RIPs on human immunodeficiency virus (HIV) and cancers. Coupling with polyethylene glycol (PEG) increases plasma time and reduces antigenicity. RIPs conjugated with antibodies to form immunotoxins increase the selective toxicity to target cells. The prospects for future development on the engineering of RIPs for improving their pharmacological properties are also discussed.

## 1. Introduction

Ribosome inactivating proteins (RIPs) are a group of cytotoxic N-glycosidases. They are mostly found from plants and a few from bacteria [1]. RIPs are classified into three types according to the number of subunits and the organization of the precursor sequences [2]. Type 1 RIPs such as trichosanthin (TCS) and momorcharin (MMC) have a single polypeptide chain with catalytic activity. Type 2 RIPs such as ricin and abrin are heterodimeric, with an active A chain linked to a lectin-binding B chain by a disulfide bond. Type 3 RIPs such as maize ribosome-inactivating protein and barley jasmonate-induced RIP (JIP60) contain a region within the protein that is removed for activation [3,4].

In general, RIPs remove a specific adenine in the α-sarcin/ricin loop (α-SRL) of rRNA, resulting in blocking the binding of elongation factor [5]. The depurination of α-SRL loop causes the GTP binding site to lose the ability to activate GTP hydrolysis. Protein synthesis is thus impeded and the cell dies [6]. Because of lectin-binding properties, most of type 2 RIPs have higher rate of cell entry and hence cytotoxicity [7]. RIPs have also been found to exhibit other enzymatic activities, including superoxide dismutase (SOD) [8], phospholipase [9] and depurination of DNA, RNA and poly (A) [10]. Several RIPs such as Momordica anti-HIV protein (MAP30) and gelonium anti-HIV protein (GAP31) exhibit a topological activity on plasmid and viral DNA, for example HIV-1 long terminal repeats (LTRs) [11]. RIPs can also induce cell apoptosis by a mechanism independent from the depurination of the SRL loop [12]. Because of their cytotoxicity, several RIPs have been tested for anti-tumor, anti-viral, anti-bacterial and anti-fungal properties. Clinical trials, such as gelonin for treating myeloid malignancies [13], ricin for treating leukemia [14] and TCS and pokeweed antiviral protein (PAP) for treating human immunodeficiency virus (HIV) [15,16] have been carried out.

Despite RIPs showing great potential in clinical applications, side effects such as inducing immune responses, short plasma half-life and non-specificity have limited their uses. Over the years, a number of works have been carried out to modify RIPs to reduce these problems. Antibodies can be conjugated with RIPs to form immunotoxins (ITs). Because of the selective function of the antibodies for targeting, ITs can achieve higher efficacy and lower side effects. Polyethylene glycol (PEG) has been used to couple with RIPs. The complex has increased molecular size. As a result, renal clearance, proteolytic degradation, immunogenicity and antigenicity are reduced.

## 2. Anti-HIV activity of RIPs

### 2.1. Anti-HIV Activity of Representative RIPs

**Figure 1:**
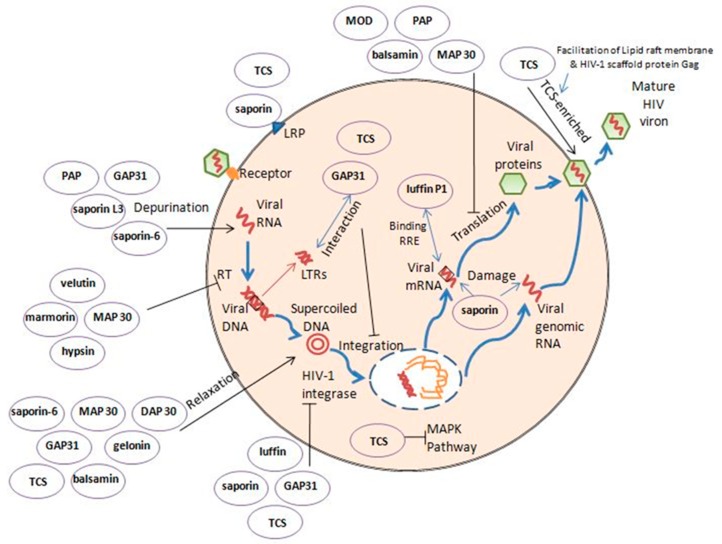
The cell cycle of HIV and the anti-HIV mechanism of representative RIPs. RIPs including TCS, GAP31, MAP30, PAP, marmorin and saporin can attack different steps of the life cycle of HIV and inhibit its growth. The mechanisms are not just due to rRNA depurination. TCS: trichosanthin; MAP30: momordica anti-HIV protein. GAP31: gelonium anti-HIV protein; PAP: pokeweed antiviral protein; MOD: maize ribosome-inactivating protein; DAP30: dianthus anti-HIV protein; MAPK: mitogen-activated protein kinase; HIV: human immunodeficiency virus.

RIPs including MAP30, saporin, TCS, gelonium anti-HIV protein (GAP31) and α-momorcharin (α-MMC) possess anti-HIV activity and other anti-viral activities. It has been found that RIPs affect the life cycle of human immunodeficiency viruses (HIV) including reverse transcription, integration, replication and assembly (Figure 1), not only due to their N-glycosidase activity.

Integration of the viral DNA plays a significant role in the replicative cycle of retroviruses. Saporin, TCS, GAP31 and luffin have all been reported possessing HIV-1 integrase inhibitory activities. 

TCS is one of the most studied RIPs, regarding its anti-HIV activity. HIV-1 integration was also inhibited by TCS, which was attributed to the temporary interaction between TCS and HIV-1 long-terminal repeats (LTRs) [17]. Some TCS mutants without anti-HIV-1 activity still had depurination activity, which meant that N-glycosidase activity may be dissociated from the anti-HIV mechanism [18]. The anti-HIV activity of TCS was eliminated by c-Jun N-terminal kinase (JNK) inhibitor CEP-11004. Jun amino-terminal kinase (JNK) is a member of stress-activated protein kinases (SAPK), which belong to the mitogen-activated protein kinase (MAPK) family. TCS exhibited anti-HIV activity and it may through the MAPK signal pathway [19]. A similar result was found when HSV-1 infected cells were treated with TCS. The p38 MAPK and B-cell lymphoma 2 (Bcl-2) induced by HSV-1 were inhibited by TCS [20]. A singular TCS hijacking HIV-1 strategy was revealed. HIV-1 scaffold protein Gag and lipid raft membrane facilitated the formation of TCS-enriched virions. The infection ability of HIV-1 with TCS was reduced dramatically [21]. Chemokine (C-C motif) ligand 5 (RANTES) and alpha-stimulated chemotaxis by stromal cell-derived factor (SDF)-1 were significantly increased by TCS, while pertussis toxin-sensitive G proteins were activated simultaneously. The activation of chemokine receptors provoked by chemokine was strengthened by TCS [22]. The relaxed circular DNA can be broken into a linear DNA by TCS, which indicated that TCS has DNase-like activity [23], which may also be a possible mechanism. Trichobitacin isolated from the root tubers of *Trichosanthes kirilowii* transiently decreased the expression of HIV-1 p24 antigen [24]. 

Saporin was found to inhibit HIV integrase 3’ end processing activity (anti-HIV-1 integrase), induce viral apoptosis and suppress HIV propagation, which are unrelated to N-glycosidase activity [25]. Saporin-6 and saporin L3 exhibit classical depurination activity targeting the GAGA conserved sequence of RNA [26]. Saporin-6 was also reported to have DNA nuclease activity [27]. Isoform saporin-L1 can inhibit viral replication which may be related to the adenosine glycosidase activity on DNA, genomic RNA and mRNA [28]. However, the anti-HIV activity of saporin-6 is found independent of its RNA N-glycosidase activity, and may be related to apoptosis [25]. Like saporin, luffin also showed HIV integrase inhibitory activities on 3′ end processing and strand-transferring, which leads to anti-HIV-1 replication [29].

MAP30 (Momordica anti-HIV protein) displays DNA glycosylase activity contributing to HIV-1 integrase inhibition. Besides, MAP30, alpha- and beta-momorcharins depress HIV replication [30]. MAP30 is also able to relax supercoiled DNA [31]. MAP30 was found suppressing the expression of HIV core protein p24 and viral-related reverse transcriptase (RT) activity without cytotoxicity and cytostaticity [32]. MAP30 can assist other anti-HIV drugs including dexamethasone and indomethacin in achieving higher efficiency [30]. 

GAP31 (gelonium anti-HIV protein of 31 kDa) and MAP30 block the infection of HIV-1 in T lymphocytes and monocytes and viral replication [11]. They also show both anti-HIV and anti-HSV activity [33]. They manifest inhibitory activity on HIV-1 integrase attributed to the topological activity toward HIV-1 long-terminal repeats (HIV-1 LTRs) [11]. GAP31 interacts with 5′ overhanging adenosine ends, but not with blunt ends, which revealed that it acts like DNA adenosine glycosidase towards the accessible adenosine [34]. A 33-aa segment (KGATYITYVNFLNELRVKTKPEGNSHGIPSLRK) of GAP31, K10-K42, was shown to be the shortest peptide that elicits anti-HIV effect [35]. 

PAP (pokeweed antiviral protein) inhibits viral protein synthesis in HIV-1 infected cluster of differentiation 4 (CD4) + T cells [36]. Engineered nontoxic PAPs, FLP-102((151)AA (152)) and FLP-105((191)AA(192)), have the potency of nucleoside reverse transcriptase inhibition toward inhibitor-resistant HIV-1 with less cytotoxicity than native PAP [37]. PAP, MAP30 and GAP31 were nontoxic to human sperm, thereby they could be applied to inactivate infective viruses and virus-infected cells in semen [38].

DAPs 30 and 32 (dianthus anti-HIV proteins, 30 and 32 kDa), as well as GAP31, are able to relax supercoiled DNA and cleave double-stranded DNA into a linear one [39].

Balsamin, purified from *Momordica balsamina*, inhibits HIV-1 replication in both human T lymphocyte cell lines and human primary CD4+ T cells [40]. Balsamin is also capable of relaxing super-coiled DNA into the linear form [41].

### 2.2. Engineering of RIPs for Improving the Anti-HIV Efficacy

Acquired immune deficiency syndrome (AIDS) patients treated with TCS showed non-dose-related reversible mental status changes including dementia, and even coma [15]. GLQ223, an inhibitor of HIV replication, is a highly purified TCS formula to treat HIV patients with higher safety than TCS. A flu-like syndrome was the major adverse effect associated with GLQ223 [42]. Different strategies were used to remit the side-effect of RIPs, such as competitive binders and steric hindrance [43]. However, immunotoxins (ITs) are more effective because of their high specificity and selectivity. RIPs, especially PAP and ricin A chain (RTA), have been utilized to make ITs for therapeutic use [43]. 

Immunotoxins (ITs) are chimeric proteins that consist of RIPs or RIP fragments and moiety for targeting [43]. Targeting moiety includes antibodies, cytokines, growth factors, hormones and lectins. ITs were first designed with whole RIP linking to full length monoclonal antibody (mAb) by disulfide bond. Type 2 RIP has lectin-binding domain (B-chain) with multiple binding sites appearance maintained at a high level of non-specific internalization. To improve the performance of the IT, B-chain of type 2 RIP was removed, or its binding sites were blocked in the second generation of ITs. The third generation ITs are recombinant immunotoxins. Recombinant RIPs genetically fused to the targeting portion of mAb by a peptide linker increases homogeneity. A single chain Fv fragment (scFv) with retained targeting ability leads to smaller size of ITs, which may affect both cell penetration and clearance. Two major drawbacks of ITs are immunogenicity and vascular leak syndrome (VLS). Human or humanized antibody formats are applied to reduce immunogenicity in the fourth generation. Antigenic epitopes modification of RIPs is also applied. ITs can be used combining with other therapeutic agents to achieve synergic effect [44,45]. 

Most anti-HIV ITs were designed targeting the HIV envelope glycoprotein and surface antigens. Several RTA based ITs with different ligands targeting to an external envelope glycoprotein (gp120) of HIV and CD4 were tested for the anti-HIV efficacy; ligands included 0.5beta, anti-gp120 and mAb 924 [46,47,48]. It was later found not much improvement was observed, while anti-gp41 (mAb 7B2) together with soluble CD4 showed anti-HIV activity [46]. Pulchellin, was conjugated to mAb 924 and mAb 7B2 for recognizing gp120 and gp41; it showed similar characteristic with RTA ITs [49]. mAb 924 and 7B2 were conjugated to RTA and pulchellin by succinimidyl 6-[3(2-pyridyldithio) propionamido] hexanoate. The lysine and N-terminus on antibody and cysteine on RTA and pulchellin were involved in this conjugation [49].

**Figure 2:**
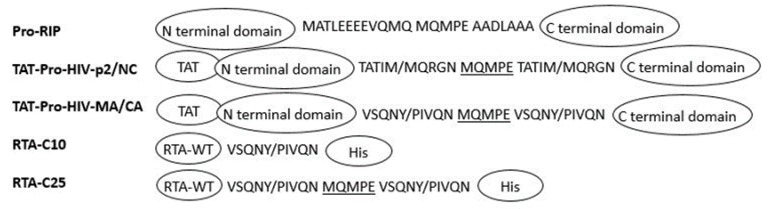
Schematic diagram of the RTA and maize RIP variants. The recombinant maize RIP precursor pro-RIP contains a 25 amino acids internal inactivation region. RTA: ricin A chain; HIV: human immunodeficiency virus; TAT: transcriptional activator Tat protein. TAT-Pro-HIV-P2/NC and TAT-Pro-HIV-MA/CA: N-termini of pro-RIP were fused with a TAT sequence. First and last 10 aa in internal inactivation region were replaced by the HIV-1 recognition p2/NC site (TATIM/MQRGN) and the MA/CA site (VSQNY/PIVQN), respectively. RTA-C10: MA/CA site fused to C-terminal of RTA. RTA-C25: two MA/CA sites were fused and separated by MQMPE (middle five residues of pro-RIP).

RTA and Maize RIP variants were linked with HIV-1 protease recognition sequences to the C-terminal or internal inactivation region (Figure 2), which could be activated by HIV-1 protease [50,51]. Maize RIP has a 25-amino acid internal inactivation region, which is able to sterically block the interaction with ribosome. This provides a switching mechanism resulting in specific targeting to HIV-infected cells with low cytotoxicity to normal cells. The internal inactivation region was replaced by HIV-1 protease recognition site. Transcriptional activator Tat protein (TAT) sequence was fused to the N-terminus. The scheme of engineering is shown in Figure 2. Recombinant active maize RIP also exerted better anti-viral activity in vivo, with the decrease of plasma virial burden transiently in chimeric simian-human immunodeficiency virus (SHIV) 89.6-infected macaque model [52]. RTA linked with HIV-1 protease recognition sequences also exhibited a more specific activity towards HIV-1 infected cells [51].

PAP was linked to mAbs targeting CD4, CD5 or CD7 antigens. The variants exerted anti-viral activity through inhibition of HIV-1 replication in HIV-1 infected CD4+ T cells and activating T cells from two asymptomatic HIV-1-seropositive patients [53].

TXU (anti-CD7)-PAP increased plasma half-life to 12.4 +/− 1.4 h and decreased systemic clearance to 2.7 +/− 0.7 mL/h/kg in adult HIV-infected patients. TXU-PAP had low toxicity. All six patients treated by 5 microg/kg dose level showed no adverse effects with viral burden reduction [16].

## 3. Anti-tumor activity of RIPs

### 3.1. Anti-Tumor Activity of Representative RIPs

TCS exerts anti-tumor activity to a wide spectrum of cancers by multiple mechanisms. The invasion, migration and epithelial-mesenchymal transition (EMT) of cervical cancer cells were inhibited, which might be relevant to the restriction of signal transducer and activator of transcription (STAT5))/C-myc signaling pathway activation by TCS. The level of B-cell lymphoma 2 (Bcl-2) and expression of antigen ki-67 associated with cellular proliferation and ribosomal RNA transcription and Phospho-c-Myc (P-C-myc) were decreased while the activation of caspase-3 was increased [54]. The apoptosis-inducing activity of TCS was attributed to the promotion of caspase-8 and caspase-9 pathways, along with the activation of caspase-3 and PARP cleavage in breast cancer cells [55]. TCS mediated Phosphoinositide 3-kinase (PI3K)/Protein kinase B (AKT) pathway and thus enhanced cytotoxicity and apoptosis-inducing activity of an anti-cancer therapy Gemcitabine against non-small cell lung cancer [56]. TCS also enhanced the cell penetration of Granzyme B leading to apoptosis of tumor cells [57]. It was shown that TCS incited autophagy in gastric cancer cells via increasing the level of autophagy protein 5 (ATG5), altering microtubule-associated protein 1A/1B-light chain 3 (LC3) I to LC3 II, inducing reactive oxygen species (ROS) and stimulating nuclear factor kappa-B (NF-κB)/Tumor protein p53 (p53) pathway [58]. ROS induction might be related to extracellular Ca^2+^, which is involved in the apoptosis of human choriocarcinoma (JAR) cells [59]. TCS was able to inhibit angiogenesis in JAR cells through the reduction of vascular endothelial growth factor and inhibition of angiogenic signal, which contributed to the anti-cancer effect [60]. Smac (a mitochondrial protein) pathway was regulated by TCS in CaSki cervical cancer cells [61]. Low-density lipoprotein (LDL) receptor-related protein 1 (LRP1) is a receptor facilitating TCS to enter JAR cells, while no significant endocytosis of TCS was found in Hela cells [62]. Notch signal was downregulated by TCS in the nasopharyngeal carcinoma (NPC) cell line CNE2 [63]. Besides tumor cell apoptosis induction and antiproliferation ability, the host immune system mediated by TCS might be another pathway for inhibition. T cells such as interferon-gamma (IFN-γ) producing CD4(+) and CD8(+) T cells, were increased by TCS in the 3LL Lewis lung carcinoma tumor model. TCS had upregulatory activity towards the expression of tumor suppressor in lung cancer 1 (TSLC1) and class I-restricted T cell-associated molecule (CRTAM) [64]. TCS exhibited antiproliferative activity on leukemia and lymphoma, which attributed to the induction of T-lymphocyte cell apoptosis and inhibition of B-lymphocyte cell growth by S-phase cell cycle arrest [65].

**Figure 3:**
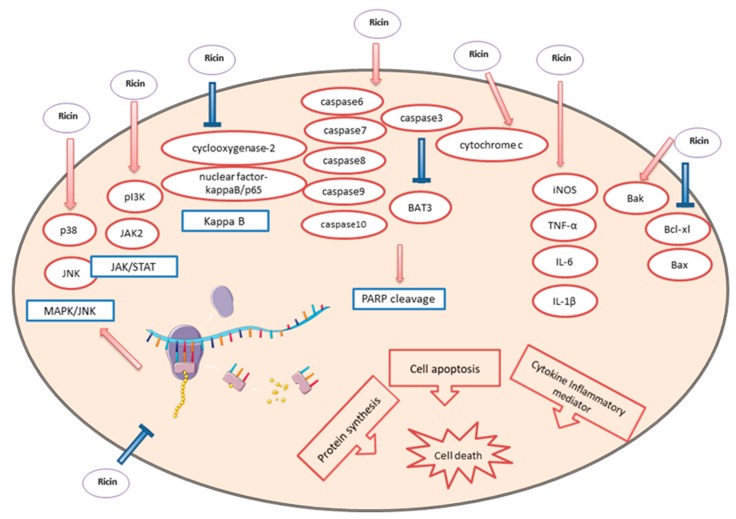
Ricin-induced cell death in the anti-tumor process. Arrows represent the activation of receptors and blunt arrows represent inhibition of receptors. Pathways involved are stated in blue boxes. The inhibition of protein synthesis, cell apoptosis and the release of cytokine inflammatory mediators are considered as the possible mechanism. p38: p38 mitogen-activated protein kinases; JNK: c-Jun N-terminal kinase; MAPK: mitogen-activated protein kinase; PI3K: phosphoinositide 3-kinases; JAK2: Janus kinase 2; STAT: signal transducer and activator of transcription; PARP: poly ADP-ribose polymerase; iNOS: inducible nitric oxide synthase; TNF-α: tumor necrosis factor-α; IL: Interleukin; Bcl-xl: B-cell lymphoma-extra large; Bax: Bcl-2-associated X protein.

Ricin also exhibits an anti-tumor property. Ricin inhibited the growth of sarcomas in rats [66], and it increased the survival rate of Ehrlich ascites tumor-bearing mice [67]. It also shows promising effect on nude mice with human xenograft [68]. A phase I clinical study on 54 cancer patients with different kinds of tumors was taken and thus confirmed its properties [69]. The inhibition of protein synthesis was first considered attributing its anti-tumor activity. Cell apoptosis and the secretion of cytokine inflammatory mediators were shown to be the related mechanisms [70,71] (Figure 3). The treatment of ricin caused the activation of p38 and jun-N-terminal kinases (JNKs) [72]. Phosphoinositide 3-kinase (PI3K) and Janus kinase 2 (JAK2) were also involved in the activation of RAW264.7 mouse macrophage cells treated by ricin toxin-binding subunit B [73]. Ricin caused proinflammatory responses on human airway cells, which was related to stress-activated protein kinases and nuclear factor kappa-B (NF-kappaB) [74]. Both two main pathways, extrinsic (receptor mediated) and intrinsic (mitochondrial pathway), were involved in the ricin mediated cell apoptosis, following the activation of poly (ADP-ribose) polymerase (PARP) [75,76]. In addition, rapid release of cytochrome c was observed in ricin treated cells [75]. Ricin has been shown to induce the secretion of proinflammatory cytokines mediator such as tumor necrosis factor alpha (TNF-α) and interleukin-1 beta (IL-1β) [77,78].

Riproximin is a type 2 RIP that up-regulated the anti-cancer cytokine IL24/MDA-7 and ER-stress-related GADD genes; it also down-regulated the genes relating to migration (RHO GTPases), anti-apoptotic activities (BCL family), and cell cycle (cyclins) in selected human breast cancer cells MDA-MB-231 and MCF-7 [79]. Similar results were confirmed by using selected human and rat colorectal cancer (CRC) cell lines [80].

The anti-cancer effect of α-MMC was tested in human breast cancer cells MDA-MB-231 and MCF-7, but the relatively high cytotoxicity limited its therapeutic use [81]. After treated with α-MMC, cytochrome c was released, and intracellular free calcium concentration was increased, and calcium overloading led to cell death [82]. c-Jun N-terminal kinases (JNKs) signal pathway, which relates to cell apoptosis, was also triggered by α-MMC [83]. Many studies demonstrated that α-MMC involved in similar pathways with TCS, such as caspase-3 and 9 activations and interaction with low density lipoprotein receptor-related protein 1 (LRP1) [83,84]. LRP1 plays a vital role in the cytotoxicity mechanism of α-MMC because α-MMC mediated cytokine expression and MAPK pathway, which would be hindered by LRP1 silencing. α-MMC inhibited immune response through the inhibition of IL-1β, IL-2, IL-8, IL-9, IL-12, MIP-1α/β, MCP-1 and elevated the expression of IL-1ra and RANTES in human monocyte THP-1 cells. The regulation of cytokine release by α-MMC revealed that α-MMC might be applied to treat tumor-associated macrophages (M2 subtypes) [85]. 

Curcin, a type 1 RIP, could inhibit the growth of several tumor cell lines at 5 μg/mL, such as NCL-H446, SGC-7901 and S180 [86]. Curcin C, which shares highly conserved sequence with curcin, elicited inhibitory activity against the osteosarcoma cell line U20S with the half maximal inhibitory concentration (IC50) value 0.019 μM when IC50 of curcin is 0.27 μM [81].

Viscumarticulatum RIP (Articulatin-D), one of the mistletoe RIPs, could selectively inhibit acute T-cell leukemia. Caspase-8 and -3 were also involved. Early signals of apoptosis induction of Articulatin-D are exposure of phosphatidylserine and increased level of mitochondrial membrane potential [87]. Aviscumine and its native form mistletoe lectin-I increased the amount of cancer cell-specific T-cells resulting in more T-cell-mediated tumor cell lysis in a mouse glioma model. The level of the proteins associated with immune response was increased [88].

### 3.2. Engineering of RIPs for Improving the Anti-Cancer Efficacy

Most RIPs ITs have shown anti-cancer potential, particularly for hematological malignancies, which are easier accessed than solid tumors [45]. The presence of clusters of differentiation (CD) on hematological cells surface are considered as ideal targets for better design of ITs [89]. 

Sap-SO6 (the main isoform of saporin) was linked to CD2, CD7, CD19 and CD22 antigens found on human leukemia and lymphoma plasma membrane surface to make ITs [90]. The saporin ITs generated increased selective cytotoxicity at least 100-fold more than saporin alone [91]. Anti CD30-Saporin was reported reducing 60% tumor mass when used to treated refractory Hodgkin lymphoma patients [86]. However, it had transient hepatotoxicity when a single dose up to 0.8 mg/kg was applied [86]. 

RTA was also used in constructing ITs; some recent studies are reviewed below. A preliminary clinical study found that BCMab1-Ra, an IT consisting of RTA and BCMab1 (a novel monoclonal antibody that specifically recognized the aberrantly glycosylated Integrin a3b1 in bladder cancer), cured a patient with multiple tumors on the bladder and achieved no tumor recurrence in 3 years [92]. RTA conjugated with anti-HER2 scFv 4D5 and the endoplasmic reticulum-targeting peptide KDEL had 440-fold increase in anti-ovarian cancer cell activity compared to RTA alone. The specificity of this IT RTA-4D5-KDEL to HER2 was high so the toxicity to normal cells was low [93]. Combotox, a 1:1 combination of anti-CD19 and anti-CD22 immunotoxins, was conjugated to deglycosylated RTA, which showed higher efficacy than either IT in pediatric precursor B-lineage acute lymphoblastic leukemia (pre-B ALL) [94]. 

Besides plant RIPs, bacterial-originated toxins such as Pseudomonas exotoxin (PE) and diphtheria toxin (DT) were also used in ITs. Denileukin diftitox (Ontak) was the first immunotoxin approved by the Food and Drug Administration (FDA), which consists of Interleukin-2 ligand and DT [95]. A number of PE-based ITs have been under clinical trials. A recent example is the antimesothelin immunotoxin SS1(dsFv)PE38 (SS1P), which is the combination of PE38 (a modified Pseudomonas exotoxin A) and a murine antimesothelin variable antibody fragment. SS1P displayed high activity against malignant pleural mesothelioma in phase I clinical trial [96]. To achieve higher efficacy, researchers applied a tumor-seeking bacterial system by engineering *Salmonella typhimurium* to make it selectively expressed and released TGFα-PE38 (transforming growth factor alpha-PE38). The released TGFα-PE38 was then tested in mice with implanted colon or breast tumor cells, which expressed high levels of EGFR (epidermal growth factor receptor). Lower solid tumor growth rate was shown comparing to just intracerebral infusion of TGFα-PE38 [97].

Vascular leak syndrome is a major side effect of many RIP-based ITs. Ricin and T22, a ligand of the cell surface marker C-X-C motif chemokine receptor type 4 (CXCR-4), were assembled to form nanostructures, which exhibited specific anti-tumor activity and avoided VLS [98].

Besides targeting CD antigen, cell penetrate peptide (CPP) was adopted to improve specificity towards cancer cells. TCS fused with heparin-binding domain (HBD), a human derived cell-penetrating peptide CPP, could increase the apoptosis rate of HeLa cells when compared with treated TCS alone [99]. It offered an efficient delivery to cancer cells. 

A co-delivery system of TCS and albendazole (ABZ) inhibited drug-resistant tumor cells (A549/T and HCT8/ADR) proliferation and tumor metastasis [100]. ABZ was covered by albumin-coated silver nanoparticles linked with low-molecular-weight protamine (a CPP) modified TCS; it could impair cytoskeleton.

## 4. Challenges in Therapeutic Applications

The immunogenicity of RIPs is an obstacle in usage. Although RIPs exhibit immunosuppressive activity [101], plant-originated RIPs readily stimulated immune system of patients, even causing allergic symptom [102]. Furthermore, the short plasma half-life of RIPs reduced drug exposure to targets, thus limiting clinical application. The plasma half-life of wild-type TCS was 9 min [103]. Repeated administration is needed to preserve the adequate level owing to renal insufficiency of small molecular weight RIPs [103]. However, repeating administration caused strong immune reaction [102]. Another side effect is neurotoxicity. TCS was examined without direct toxicity to the central nervous system (CNS). However, HIV-infected patients treated by TCS were reported to have adverse CNS reactions [104]. The HIV-infected macrophages might be altered by TCS treatment, which aggravated neurological symptoms [104]. Solid tumor mass is hard to access, leading to reduced efficacy. Intracavitary therapy with ITs might solve this problem [105]. Several studies have been carried out to enhance the pharmacological properties of RIPs.

## 5. Coupling with Polymer Polyethylene glycerol (PEG) and Dextran

PEGylation is a common strategy used to improve drug performance. PEG is biocompatible with high hydrophilicity, low toxicity and non-immunogenicity [106]. After coupling, the size and molecular weight are increased to avoid rapid renal clearance and proteolytic degradation for longer half-life. Antigenicity and immunogenicity can be decreased, while pharmacokinetics and pharmacodynamics can be improved. Moreover, permeation retention effect was increased by PEGylation, which helped to target tumoral tissues [107]. Non-specific PEGylation was applied at first. Although reactive amino acids such as cysteine, arginine and serine can be chosen [108], site-directed conjugation has provided better achievements. The antigenic sites are mapped and then antigenicity can be alleviated through PEGylation, while the original enzymatic activity could be least affected. For successful conjugation, a site extending from the protein surface is preferred [109]. Many RIPs PEGylations were attempted to advance their pharmacological properties (Table 1).

Gelonin (GAP31) was covalently coupled to methoxypoly (ethylene glycol) (mPEG) 2000, mPEG 5000 and mPEG succinimidyl succinate 20K (SS-20PEG). mPEG does not affect the positive charge of protein, while charges alteration may result in lowering biological activity. The plasma half-life time of all conjugations above was increased. PEGylation also decreased organ uptake. Coupled of mPEG retained immunogenicity, while SS-20PEG conjugated decreased cytotoxicity [110].

Site-directed PEGylation of TCS showed that PEG 20,000 is better than PEG 5000. Coupled to PEG 20000, the plasma half-life extended due to the enlarged size and resistance to proteolysis. Immunoglobulin G (IgG) level was also reduced because of decrease in immunogenicity [118]. The IgE level was reduced but the IgG level was maintained when conjugated to PEG 5000 [109,118]. Because tremendous liver uptake is through carbohydrate-mediated recognition, carbohydrate-directed PEGylation can improve the pharmacokinetics of RTA. Meanwhile, the circulation time was increased and antigenicity was reduced by masking the epitopes. This makes carbohydrate-directed PEG-RTA conjugate a potential anti-tumor drug [120]. Coupling PEG to maize RIP (MOD) gave similar results, including pharmacokinetics improvement and antigenicity reduction [109]. Mono-, di-, tri-PEGlyated α-MMC and MAP30 dramatically reduced the immunogenicity and maintained biological activities [112,113]. The enzymatic activity assay showed that mono-PEGylated α-MMC strongly inhibited the growth of human cervix adenocarcinoma cells [116]. Sun et al. provided a method to isolate high purity mono-mPEGylated MAP30 and α-MMC, which can be useful for further RIP drug examination [111]. In vivo study implied that the hepatic toxicity of α-MMC was reduced after PEGylation [115]. The plasma half-life of α-MMC was sharply increased from 6.2–7.5 h to 52–87 h [114]. The homogeneity of PEGlyated α-MMC was further improved by site-specific conjugation of mPEG-ALD [116]. Due to steric hindrance of active sites, PEGylation may lead to biological activity reduction, which can be compensated by longer plasma half-life [119].

Coupling dextran on RIPs was also studied to improve their performance on anti-cancer and anti-HIV. Similar to PEGylation, dextran can increase circulation time and reduce IgG or IgE responses [103,121,122]. Dextran may be used as a linker to connect monoclonal antibodies and RTA, and to improve selectivity toward target cells [123]. A lower rate of plasma clearance prolongs the plasma half-life of TCS after coupling to dextran T40 by a dialdehyde method [103]. The toxicity and potency were decreased [124]. TCS coupled with dextran had the IgE level reduced eight times compared to wild-type [121] To reduce the antigenicity, TCS was also coupled by bromodextran T20 [125]. To obtain better conjugate, potential antigenic site K173 of TCS was mutated to cysteine and coupled with dextran. Site-directed conjugate dextran-K173C had the hypersensitivity reaction and the level of IgG andIgE decreased [126].

## 6. Perspectives

The therapeutic applications of RIPs, in particular anti-HIV and anti-tumor potential were exploited in the last several decades [43]. The main obstacles of utilization include short plasma half-life, non-selective cytotoxicity and antigenicity. Most PEGylation increases plasma time and reduces antigenicity. RIPs conjugated with antibodies to form immunotoxins increase the selective toxicity to target cells.

Immunotherapy can assist chemotherapy to improve efficacy [127]. An IT is co-applied with a small molecular drug or another IT to achieve high efficacy and reduce side effects. Anti-CD3 and anti-CD7 were conjugated separately to RTA and mixed to treat steroid-refractory acute graft-versus-host-disease. The mixture exerted higher efficacy than one alone with low side effects in clinical trial I/II [128]. Saponin, which is generally classified as steroidal or triterpenoidal, can act as an endo/lysosomal escape enhancer. It was commonly used together with type I RIP to facilitate escape of RIP from endo/lysosomal degradation in order to increase efficacy of RIP or ITs [43].

“Cocktail therapy” is an effective strategy to increase the effectiveness of ITs [129]. The synergistic effect can be achieved when two or even more suitable ITs corporate. Lung cancer cells can easily generate resistance against tumor necrosis factor-related apoptosis-inducing ligand (TRAIL) [130]. TCS was found inducing TRAIL sensitivity of non-small cell lung cancer (NSCLC) by regulating death receptors and proteins involved in invasion and cell cycle [130].

A number of RIP-derived drugs reached clinical trial, but then failed due to severe side effects and little efficacy (Table 2). At present, an RIP diphtheria toxin (DT) derived drug denileukin diftitox (Ontak) has been approved by FDA for treating cutaneous T-cell lymphoma (CTCL).

With the rapid development of immunotoxin engineering technology, it is possible to acquire RIP-based drugs with high efficacy and low side effects. A list of endosomal escape enhancers such as saponin, TAT (transcriptional activator Tat protein), perforin and ricin B-chain were observed to facilitate RIP [43]. mAbs help increase the specificity; adequate combination of ITs with different mAbs can achieve better curative effect [96]. Novel technology has been tested, such as photochemical internalization (PCI). PCI is a light-based method and is employed to trigger the endosomal escape of RIP. Saporin linked with a photosensitizer functionalized CPP showed cytotoxicity augmentation in MC28 fibrosarcoma cells [135]. Another study also showed that the cytotoxicity of IT 225.28-saporin was strengthened by using PCI with a photosensitizer disulfonated tetraphenyl chlorin (TPCS2a) [136]. TCS was conjugated with an albumin-binding domain and a legumain-substrate peptide as a modified IT for better delivery efficiency, which can make use of the nutrient transporter pathway of albumin-binding proteins. Protease legumain at the tumor sites can dissociate TCS from an albumin-binding domain, which gives a new strategy for tumor therapy [137]. These new research findings provide RIP therapy a promising future.

## Figures and Tables

**Table 1:** Representative PEGylated RIPs.

RIP	Type	PEG	Disease	Site	Ref.
Gelonin (GAP31)	1	methoxypoly(ethylene glycol) (mPEG)2k/mPEG5k/mPEG succinimidyl succinate20k (SS-20PEG)	Tumor and HIV	Random	[110]
Alpha-Momorcharin (α-MMC)	1	mPEG-succinimidyl carbonate (mPEG-SC)10k	Tumor	N-terminal	[111]
α-MMC	1	20 kDa (mPEG)2-Lys-NHS	Tumor	Mono-, di-, tri- PEGylated	[112], [113], [114], [115]
α-MMC	1	20 kDa mPEG-butyraldehyde (mPEG-ALD)	/	N-terminal	[116]
Momordica anti-HIV protein (MAP30)	1	mPEG-SC10k	Tumor	N-terminal	[111]
MAP30	1	20 kDa (mPEG)2-Lys-NHS	Tumor	Mono-, di-, tri- PEGylated	[113]
Trichosanthin (TCS)	1	PEG5k	/	YFF81-83ACS/ KR173-174CG/[YFF81-83ACS, KR173-174CG]	[117]
TCS	1	PEG5k	HIV-1	Q219C/K173C/S7C	[118]
TCS	1	PEG5k/PEG20k	/	Q219C/K173C/S7C/[K173C, Q219C](KQ)	[119]
Ricin A Chain (RTA)	2	PEG2k/mPEG2k	/	Random	[99]
RTA	2	Monomethoxy-PEG hydrazide (mPEG-HZ)5k/monomethoxy-PEG succinimidyl propionate (mPEG-SPA)5k	Tumor	Carbohydrate/Amine- specifically	[120]
Maize RIP (MOD)	3	PEG5k/PEG20k	HIV, Chinese rhesus macaques	K78C/K264C	[109]

**Table 2:** Recent (after 2000) representative ricin & RTA immunotoxins tested.

Immunotoxin	Toxin	Ligand	Target Antigen	Disease	Clinical Trial Status	Reason for Suspension	Ref.
anti-B4-bR	ricin	Anti-B4	CD19	B-cell lymphoma	III	no differences between event-free survival and overall survival	[14]
N901-bR	ricin	N901	CD56	small-cell lung cancer	II	vascular leak syndrome	[131]
Anti-CEA-bR	ricin	I-1	carcinoembryonic antigen	hepatic metastases	III	no obvious changes in the growth rate of injected lesions	[132]
Ki-4.dgA	RTA	Ki-4	CD30	refractory CD30+ Hodgkin’s and non-Hodgkin’s lymphoma	I	vascular leak syndrome; low tolerance	[133,134]
